# Resolution of the paradox of the diamagnetic effect on the Kibble coil

**DOI:** 10.1038/s41598-020-80173-9

**Published:** 2021-01-13

**Authors:** Shisong Li, Stephan Schlamminger, Rafael Marangoni, Qing Wang, Darine Haddad, Frank Seifert, Leon Chao, David Newell, Wei Zhao

**Affiliations:** 1 Department of Engineering, Durham University, Durham, DH1 3LE UK; 2 National Institute of Standards and Technology, Gaithersburg, 20899 USA; 3 Department of Electrical Engineering, Tsinghua University, Beijing, 100084 China

**Keywords:** Physics, Applied physics, Quantum physics, Techniques and instrumentation

## Abstract

Employing very simple electro-mechanical principles known from classical physics, the Kibble balance establishes a very precise and absolute link between quantum electrical standards and macroscopic mass or force measurements. The success of the Kibble balance, in both determining fundamental constants (*h*, $N_A$, *e*) and realizing a quasi-quantum mass in the 2019 newly revised International System of Units, relies on the perfection of Maxwell’s equations and the symmetry they describe between Lorentz’s force and Faraday’s induction, a principle and a symmetry stunningly demonstrated in the weighing and velocity modes of Kibble balances to within $1\times 10^{-8}$, with nothing but imperfect wires and magnets. However, recent advances in the understanding of the current effect in Kibble balances reveal a troubling paradox. A diamagnetic effect, a force that does not cancel between mass-on and mass-off measurement, is challenging balance maker’s assumptions of symmetry at levels that are almost two orders of magnitude larger than the reported uncertainties. The diamagnetic effect, if it exists, shows up in weighing mode without a readily apparent reciprocal effect in the velocity mode, begging questions about systematic errors at the very foundation of the new measurement system. The hypothetical force is caused by the coil current changing the magnetic field, producing an unaccounted force that is systematically modulated with the weighing current. Here we show that this diamagnetic force exists, but the additional force does not change the equivalence between weighing and velocity measurements. We reveal the unexpected way that symmetry is preserved and show that for typical materials and geometries the total relative effect on the measurement is $\approx 1\times 10^{-9}$.

## Introduction

Most human activities, especially science, industry, and trade rely on measurements. The importance of measurement to global society is such that the International System of Units (SI) was created as early as 1875 so that all measurements might be traceable to a single compact set of common standards. For a long historical period the SI standards were formulated by artifacts (man-made or using a property of nature), specific objects preserved in a single location, with limited access. Undeniably inaccessible, the value that such an artifact standard realizes may also vary over time^1^, introducing dark uncertainties for precision science and high-accuracy engineering^2^. Consequently, alternatives to artifact standards have been sought since the beginning of the SI^3^. The first success was measurement by counting events of microscopic particles (e.g., atom, electron, photon, etc), first used in time measurements based on atomic clocks, which opened the door for the quantum measurement of things^4–7^. On May 20, 2019, a new International System of Units, in which all seven base units are defined by physical constants of nature, was formally adopted^8,9^ and our daily measurement activities have entered into a quantum era. With this quantum revolution of the SI, our measurement system relies now on fundamental constants which are woven into the structure of our universe and are here for all times and for all people, and are no longer tied to physical objects with limited stability and availability. The new SI provides a highly accurate or ultra-sensitive measurement foundation to support explorations that were not possible in the past^10,11^. The change is most profound for mass quantities, where the quest for an atomic or quantum based standard of mass vexed researchers for decades.

To realize the unit of mass at the kilogram level from atomic or quantum standards, two complementing technologies were eventually found, the X-ray crystal density (XRCD) method^12^ and the Kibble balance^13^.The XRCD method relies on the mass of the electron, which is given by the Rydberg constant and defined fundamental constants. Using mass spectroscopy and scaling that takes advantage of a nearly perfect single crystal silicon sphere, the electron’s mass can be scaled thirty orders of magnitude to the kilogram level with a relative uncertainty of $1\times 10^{-8}$^14^. The realization of the kilogram via the Kibble balance relies on the perfect symmetry of Maxwell’s equations and can reach a similar uncertainty^15,16^, thanks to some Nobel prize winning quantum physics^17,18^.

In the 1980s, the discovery of the quantum Hall effect by Prof. von Klitzing^19^ provided a catalyzing piece in the quest of a quantum mass standard. It was almost immediately recognized that the quantized resistance standard^20^ that resulted from von Klitzing’s work could be combined with the Josephson effect that had been theoretically postulated in 1962^21^ and experimentally verified a year later^22^ allowing the measurement of electrical power solely based on quantum effects. Once electrical power could be measured via quantum standards, a machine that precisely compares electrical to mechanical power would allow the quantum realization of mechanical power given by force times velocity. Velocity is easily measured as a unitless fraction of speed of light and the force could be, for example, the weight of a mass standard in the gravitational field of the Earth. All that is needed is a precise tool that can compare mechanical to electrical power.

Luckily, such a tool, a comparator, existed. It was proposed in 1976 by Dr. Bryan Kibble^23^, a metrologist at the National Physical Laboratory in the United Kingdom. Kibble’s invention was initially named a watt balance, emphasizing that it compares mechanical to electrical power, since the watt is the unit of power, both electrical and mechanical. Kibble passed away in 2016, and the watt balance was renamed Kibble balance to honor his contributions to metrology. The core of Kibble’s idea lies in a symmetry of electromagnetism, described by Maxwell’s equations^24^. In a nutshell, it can be described as follows: The energy of a current-carrying loop (a coil with one turn) in a magnetic field is given by the product of current, *I* and the magnetic flux, $\phi$ threading the coil. The Lorentz force in the vertical direction $F_z$ on the coil is the negative derivative of the energy of this loop with respect to its vertical position, *z*.

1 $$\begin{aligned} F_z = -\partial _z \phi I. \end{aligned}$$

In this text, we use the abbreviation $\partial _z A:=\frac{\partial A}{\partial z}$ for the partial derivative of a quantity *A* with respect to *z*. The current is easy to measure, but not the derivative of the flux through the loop. Here is where the symmetry of nature comes to the rescue: Moving the coil in the magnetic field produces an induced electro-motive force between both ends of the coil. By Faraday’s law of induction, the induced voltage, *U* is proportional to the product of the derivative of the flux times the vertical velocity, $v_z$, of the wire loop.

2 $$\begin{aligned} U = -\partial _z \phi v_z. \end{aligned}$$

Both equations can be combined to obtain the watt equation that shows the equivalency of mechanical to electrical power, and conveniently the hard to measure flux derivative vanishes.

3 $$\begin{aligned} F_z v_z = U I. \end{aligned}$$

By using the weight of a mass $F_z=mg$ for the force and the quantum measurement of the electrical power $UI=C f^2 h$, where *f* is the frequency that is used to drive the programmable Josephson junction voltage array and *C* is a known constant that depends, for example, on how many Josephson junctions are used, the mass can be written as

4 $$\begin{aligned} m = \frac{Cf^2{h}}{g{v_z}}. \end{aligned}$$

**Figure 1:**
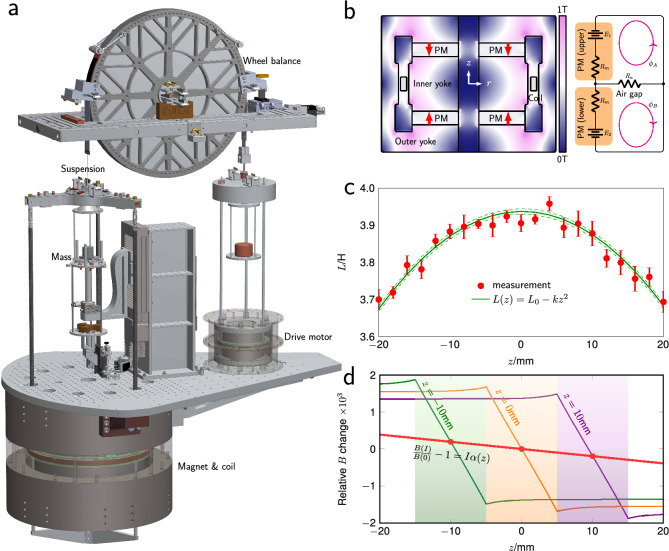
The magnet system in a Kibble balance and the coil-current effect. (**a**) presents the major elements in the fourth generation Kibble balance experiment at NIST. The left subplot of (**b**) is the sectional view of a typical permanent magnet system with symmetry, where the color map denotes the *B* field distribution. The right subplot presents an equivalent electrical circuit of the air-gap type magnet system, where $R_m$ is the magnetic reluctance of the permanent magnet, $R_a$ the magnetic reluctance of the air gap, $E_1$, $E_2$ respectively the magnetomotive force of upper and lower magnets. (**c**) shows a typical measurement of the coil inductance (frequency extrapolated to DC) as a function of coil vertical position *z*. With a up-down symmetrical magnet, it can be written as $L=L_0-kz^2$. (**d**) shows the relative magnetic field change due to the coil current in such magnet systems. The plot shows the magnetic field with a plus current $I_\text{off}$, which produces 4.9 N magnetic force. The red curve is an average magnetic field for the coil, and this field slope has been verified at BIPM as $\frac{B(I)}{B(0)}-1=I\alpha (z)$, where $\alpha$ is a linear function of *z*. Note that the field distribution with $I_\text{on}$ is an image of $I_\text{off}$ symmetrical to *B*(0).

Figure 1a shows a typical Kibble balance. Two large components are apparent: the magnet and the wheel. The wheel is a particular choice for a part that can be used as a moving and weighing mechanism. The wheel allows the comparison of electromagnetic force and mass weight while also providing the coil’s motion needed for the velocity mode. Up to the 2000s, several different types of magnet systems were used^25–29^. Over time, the field matured, and the magnet systems’ design converged to what is known as the air-gap type, yoke-based magnetic circuits^30–37^. Figure 1b shows a typical construction of such a permanent magnet system. The permanent magnetic circuit’s significant advantage is that it can supply a strong (several tenths of a tesla), uniform, and robust magnetic field without an active energy source.

While the description using the derivative of the flux is accurate and was used initially by Kibble, these days, the researchers use a different description of the same numerical quantity, the so-called geometric factor, or flux integral. The geometric factor is obtained by integrating the horizontal component of the magnetic flux density *B* that is perpendicular to the wire with a length *l* that forms the coil. It is abbreviated as *Bl*, and by virtue of Green’s theorem it is the same as $\partial _z \phi$. For the rest of the article, we consider the possible causes and consequences of inevitable imperfections in the symmetry, so that there are two different geometric factors, one for weighing mode, $(Bl)_w$, and one for velocity mode, $(Bl)_v$. A succinct equation for the relationship of both geometric factors was suggested by Robinson^38^. The widely accepted equation is

5 $$\begin{aligned} (Bl)_w=(Bl)_v(1+\alpha I+\beta I^2), \end{aligned}$$

where *I* is the current circulating in the coil during weighing mode. Observe that the current dependence of $(Bl)_w$ is canceled to first order through a common reversal trick in the design of the weighing mode: Two measurements must be made, one without and one with mass on the mass pan of the balance. The balance, however, can be biased with a tare weight, $m_t\approx m/2$, such that the currents in the coil have the same absolute value but opposite signs. The forces on the balance for the two states are

6 $$\begin{aligned} {\left\{ \begin{array}{ll} \text{mass-on}:I_\text{on}(Bl)_v(1+\alpha I_\text{on}+\beta I_\text{on}^2) +mg = m_t g\\ \text{mass-off}:I_\text{off}(Bl)_v(1+\alpha I_\text{off}+\beta I_\text{off}^2) = m_t g. \end{array}\right. } \end{aligned}$$

The tare weight is adjusted such that the currents are symmetric, $I_\text{on}=-I_\text{off}$, and it is sufficient to work with the variable $I:=I_\text{off}$. By subtracting the mass-off equation from the mass-on equation in (6), the mass can be obtained as

7 $$\begin{aligned} m =\frac{(Bl)_v}{g} \left( 2 I + 2 \beta I^3 \right) , \end{aligned}$$

where as mentioned before $(Bl)_v$ is obtained from the velocity mode. By using symmetric currents, all terms containing $\alpha$ vanish. The only remaining systematic term, $2\beta I^3$ is very small, $2\beta I^3/(2I)\approx 10^{-9}$^39,40^. Although the term is small, it is measurable by using different mass values on the Kibble balance, e.g., *m*/2, *m*, 2*m*. This process is possible in the new SI, because multiple and sub-multiples of masses can be generated without having to resort to Kibble balances using a classical scheme to subdivide masses^15,16^. In summary, the Kibble principle is preserved when symmetric currents are applied during weighing mode, because the dominant term of the dependence of the magnetic field on the weighing current drops out. The next to leading order effect is small and can be compensated for using ancillary measurements. For the remainder of this manuscript, we assume $\beta =0$ without altering the main conclusion, but simplifying the equations.

We next take up a question that has vexed the Kibble balance community for years. What if extraneous magnetic forces act on the weakly magnetic materials of the coil? Put another way, what if the coil is a magnet? We use the term weakly magnetic materials for materials that exhibit diamagnetic or paramagnetic behaviour, in other words material whose magnetization, $M=\chi H$ depends linearly on the applied external field *H*. The proportionality factor is given by the volume susceptibility $\chi$ and is negative for diamagnetic and positive for paramagnetic materials. It is impossible to build a coil without using weakly magnetic materials. The magnet wire used to wind the coil is made from copper which is diamagnetic with $\chi \approx - 10^{-5}$. The diamagnetic force has been impressively demonstrated by levitating a diamagnetic object, e.g. graphite^41^, organics^42^, water^43^, living cell^44^, even a frog^45^, in a magnetic field. The force on a very small element with volume *V* of weakly magnetic material in the air gap of a permanent magnet is given by

8 $$\begin{aligned} F_\chi =\frac{\chi V}{\mu _0}B \partial _z B. \end{aligned}$$

There is a constant static force acting on the coil, but it is common to the mass-on and mass-off measurement, similar to the coil’s weight, and it will drop out in the difference of the mass-on and mass-off measurement. To be clear, the diamagnetic force on the coil is well-known but is thought to drop out in the reversal of the current in force mode. The systematic described below is named the diamagnetic effect in force mode. It is the diamagnetic force that does not cancel between the two measurements in force mode.

Nevertheless, a systematic bias,the diamagnetic effect, cannot be ruled out, because *B* is not a constant, rather it is a function of current in the coil according to Eq. (5). Consequently, $F_\chi$ must be a function of current also. The difference between mass on and off would be

9 $$\begin{aligned} \Delta F_\chi = \frac{2 \chi V}{\mu _0}I \partial _z \left( B_v^2 \alpha \right) \end{aligned}$$

so that the quadratic nonlinearity no longer cancels and we are forced to consider $$\alpha $$. Up until 2017, $$\alpha $$ was assumed to be constant, independent of the coil position, and dispensed with. A notable article published that year associated $$\alpha $$ with the reluctance effect^46^. The reluctance effect can be explained by considering the magnetic energy stored in the magnetic field surrounding the coil due to the constant current during weighing, $$E=1/2\,LI^2$$ where *L* is the self inductance of the coil in its surroundings. Once again, a force arises as a result and in the direction of any gradient of the magnetic energy. The vertical component of this force can be written as $$F_z=-1/2\,I^2\partial _z L$$. The force points toward the maximum of the inductance, usually at the middle of the symmetry plane of the coil magnet system. This principle is well known from solenoid actuators, where an iron slug is retracted into a solenoid when it is energized with current. Here, the slug (the magnet and yoke material) is fixed, while the coil is free to move in the *z* direction. The inductance *L*(*z*) depends mostly on the symmetry of the shape and magnetic properties of the yoke and not on the permanent magnet material. For an ideal yoke $$ L =L_0-kz^2$$ is a quadratic function of *z* with $$z=0$$ in the symmetry plane of the yoke, see Fig. 1c. Interestingly, the reluctance force can be interpreted as a force produced by an additional magnetic field, so instead $$F_z=-1/2\,I^2\partial _z L = (Bl)_\text{add} I $$, and hence $$(Bl)_\text{add}=-1/2\,I\partial _z L$$. As described in Fig. 1d, experiments at the BIPM prove that this additional magnet field does, in fact, exist^46,47^. Hence, the parameter $$\alpha $$ introduced in Eq. (5) can be written as $$\alpha = -k z/(Bl)_v$$.

The partial derivative in Eq. (9) can be rewritten as $$\partial _z B_v^2\alpha = \alpha \partial _z (B_v^2) +B_v^2 \partial _z \alpha $$. The magnet systems for the Kibble balances are often designed such that $$\partial _z B_v=0$$ rendering the first term insignificant. The second term evaluates to $$-B_v^2 k/(Bl)_v $$, and the relative size of the effect can be obtained from Eq. (9) as

10 $$\begin{aligned} \frac{\Delta F_\chi }{mg} = -\frac{\chi }{\mu _0}\frac{A_c}{Nl} k. \end{aligned}$$

Here, we are formulating the effect on the wire while considering multiple turns, so the volume of the wire, *V* has been replaced by the product of the wire cross sectional area $A_c$, the length *l* and the number of turns *N*. The derivation will also work for non-current carrying elements, like the coil former or structures mounted on the coil, but the equations are more insightful for the wire. The relative effect consists of three factors and typical values are $\chi /\mu _0 ={-8}\hbox { m H}^{-1}$, $A_c/(Nl)={200}\,\hbox {mm}^{2}/(1057\cdot {834}\,\hbox {m})=2.27\times ^{-10}\hbox {m}$, and $k={550}\,\hbox {H}/\hbox {m}^{2}$. Multiplying the three factors together yields a relative force of $1\times 10^{-6}$. An amount that is more than 100 times larger than the combined relative uncertainty reported by the best experiment in the world.

Here we reach an impasse. The paradox. On the one hand, the above summary of current reasoning, modeling and experimentation supports the conclusion that the diamagnetic effect in force mode does exist. On the other hand, measurements of the Planck constant using two completely different methodologies (XRCD and Kibble balance) agree to within $1\times 10^{-8}$, supporting the conclusion that it doesn’t. Where is the truth?

A possibility that must be considered is that there is a common bias, or intellectual phase-lock among the experiments. After all, the highest precision Kibble balances share similar design parameters, and the community was driven by a common goal to seek a consensus value. Perhaps the relative size of the effect does not vary much from balance to balance. Being common mode to all, it would not be observed. But values of the Planck constant were compared among all Kibble balance and XRCD methodologies. To support such a bias among the balances requires intellectual phase lock across the competing methods and multiple laboratories on a global scale. This seems highly unlikely in a metrology community fiercely committed to objectivity.

Another possibility that must be considered is that the diamagnetic effect in force mode doesn’t exist. The deniers of this effect likened the force produced by it to the fictional force that Baron Munchausen used to pull himself out of a mire by his own hair—clearly in violation of Newton’s third law. They argue, that the current in the coil cannot exert an additional and current dependent force on itself. This force, however, is between the magnet system, altered by the current, and the coil, similar to the reluctance force that undoubtedly exists (A detailed analysis can be found in the Supplementary Information). Given the state of knowledge, it seems logical to suggest an experiment be performed to measure the effect directly. Unfortunately, this is exceedingly difficult. According to Eq. (10) the effect depends only on variables that are, for the most part, impossible to modify for a given Kibble balance. These are instruments designed to maintain absolutely constant physical, magnetic, and electrical geometries save for one coordinate. Changing the mass, and hence the current in the coil, will not change the relative contribution of the diamagnetic force. The only variable sometimes available is the coil geometry $A_c/(Nl)$, but even that is not simple. Several Kibble balances have multiple coils wound on a single former, and the Kibble experiment can be performed with different coils or different coil combinations. Unfortunately, the relative contribution of the diamagnetic effect does not change as long as all coils are immersed in magnetic flux produced by the same magnetic system, regardless if they are active (used in the experiment) or not. In summary, it is conceivable that a relative bias as large as $1\times 10^{-6}$ exists in all Kibble balance experiments.

In this article, we will solve the paradox of the diamagnetic effect in force mode. The surprising result is that the diamagnetic effect exists, but we find a symmetric effect in the velocity mode. By combining the measurements taken in velocity mode with those made in weighing mode, the bias introduced by the diamagnetic effect is canceled. These counteracting biases explain the paradox, restore confidence in the foundation of the new SI mass, and have never been described in the literature. The result is simple and satisfying: the symmetry of the Kibble balance experiment once again self corrects, and the diamagnetic effect vanishes in the combined result. This new finding will relax the requirements on the materials that the coil and components attached to it are made from. Weakly magnetic materials can be used in these cases. Still, one has to be careful not to use ferromagnetic materials, because materials with a nonlinear response to the external field are not covered by this symmetry.

## Results

### Analytical result of the diamagnetic effect in velocity measurement

In the previous section, we have argued that the diamagnetic effect exists and that it produces a large relative bias in the weighing mode of Kibble balances. The bias is so large that Kibble balances would not be able to make precise measurements. Here we show that the bias in the weighing mode is cancelled by an identical bias in the velocity mode and the Kibble principle holds.

We start by rewriting the self inductance of the coil *L*(*z*) with *N* turns according to the derivation in the “Methods” section. In a cylindrical air-gap with a mean radius $r_a$, a radial width of $w_a$, and a height $2h_a$, the inductance is given by

11 $$\begin{aligned} L(z)=L_0-\pi \mu _0 N^2\frac{ r_a}{w_a} \frac{z^2}{h_a} \;\;\Longrightarrow \;\; k=-\frac{1}{2}\frac{\partial ^2 L}{\partial z^2} = 2\pi \mu _0 N^2 \frac{r_a}{A_a}, \end{aligned}$$

where $A_a=2w_ah_a$ denotes the cross-sectional area of the air gap. By employing a cross sectional area for the coil, the relative size of the diamagnetic effect can be written compactly as

12 $$\begin{aligned} \frac{\Delta F_\chi }{mg} = -\frac{\chi }{\mu _0}\frac{A_c}{Nl} \frac{2\pi r_a \mu _0N^2}{A_a}=-\chi \frac{A_c}{A_a}\frac{r_a}{r_c}. \end{aligned}$$

**Figure 2:**
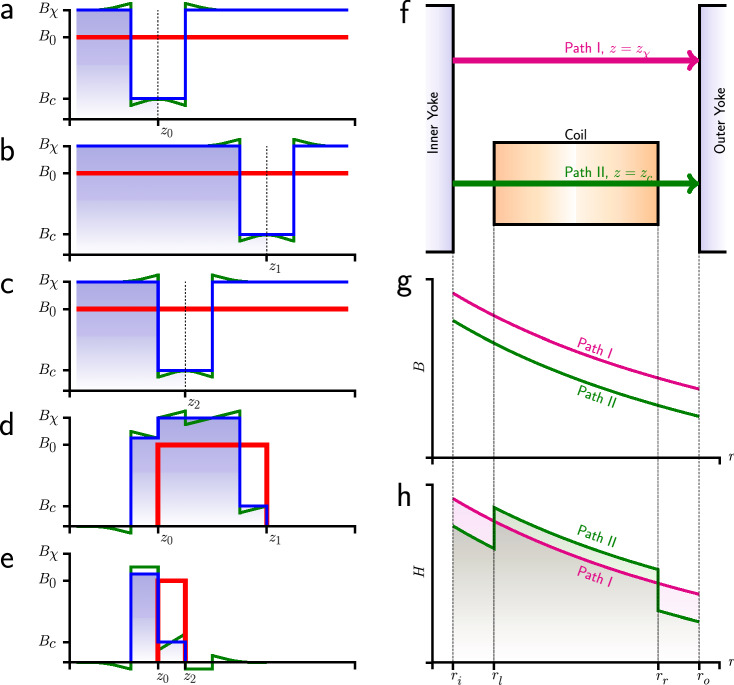
A qualitative illustration of the magnetic field distribution at different coil positions. (**a**)–(**c**) show the $B(r_c, z)$ curves at three different vertical position $z_0$, $z_1$ and $z_2$. The red curves are the magnetic profile when the coil susceptibility is zero. The blue curves are the first order approximation of *B* field curve with a diamagnetic coil, $\chi <0$. The green curves are profiles of the diamagnetic coil with higher order approaching. (**d**) and (**e**) present the *B* field difference under two configurations: $(z_1-z_0)>2h_c$ and $(z_2-z_0)<2h_c$ (coil region overlap). (**f**) show two paths horizontally across the air gap, respectively at $z_\chi$ and $z_c$. (**g**) and (**h**) present the *B* field and the *H* field distributions along two paths.

Next, we investigate what happens when a diamagnetic material is introduced to the air gap. The left plots in Fig. 2 show the magnetic flux density as a function of vertical position. Before the material is introduced, the flux density is constant throughout the gap (red line). A constant flux density for the air gap is assumed to keep the explanation simple, but is not necessary for the theory to work. Adding the coil, here with $\chi <0$, changes the flux profile. A perfectly nonmagnetic coil would have no effect, but the vertical section occupied by the coil now restricts the flux, due to the increased magnetic reluctance of the diamagnetic material in that part of the gap. The total flux produced by the permanent magnet redistributes itself, and, as a result, the flux density in the empty space increases in direct proportion to the reduction of flux through the space occupied by the coil.

For $\chi <0$, compared to the situation without the coil, $B_0$, the value of the flux density is lower at the coil ($B_c$) and higher in the rest of the gap ($B_\chi$). In the physical system, there are nonlinear effects near the edges, shown by the green curves in Fig. 2. Again, these are not important for the simplified explanation of the effect and can be ignored.

The magnetic flux threading through the coil can be obtained as the integral from the bottom of the air gap to the middle of the coil, indicated by the blue shaded region for the coil in three different vertical positions in Fig. 2a through c.

As mentioned above, the induced voltage in velocity mode is proportional to the derivative of the magnetic flux through the coil with respect to time. The flux for the baseline position of the coil $z_0$ is shown in (a), while the flux for the positions $z_1$ and $z_2$ are shown in (b) and (c). The difference in flux with respect to the coil at baseline for these positions is depicted in (d) and (e), respectively. We assume that the coil moves through the gap along a fixed trajectory with the same constant velocity in the *z* direction for the case when coil susceptibility is zero, and then again when it is $\chi$. The relative difference of the flux density change between these scenarios, and, hence, the induced voltage is given by

13 $$\begin{aligned} \frac{\Delta U_\chi }{U}=\frac{B_\chi }{B_0}-1. \end{aligned}$$

The flux density $B_\chi$ can be calculated assuming that the total magnetic flux through the air gap remains the same. At $r=r_c$, the flux integration vertically through the whole air gap can be written as

14 $$\begin{aligned} 2h_aB_0=2h_cB_c+(2h_a-2h_c)B_\chi \; \Longrightarrow \; \frac{ \left( B_\chi -B_0\right) }{\left( B_c-B_0\right) } = - \frac{2h_c}{2h_a-2h_c}, \end{aligned}$$

where the negative sign indicates that $B_\chi >B_0$ and $B_c<B_0$ when diamagnetic material is introduced. For paramagnetic material, $B_\chi <B_0$ and $B_c>B_0$.

The ratio of the change from $B_0$ of $B_\chi$ and $B_c$ to $B_0$ is identical to the ratio of the height of the occupied gap to the height of the empty air-gap, since $2h_a$ and $2h_c$ denote the height of the air gap and the coil, respectively.

In an actual magnet system, the magnetic height of the air gap $2 h_a$ differs from the geometrical height of the air gap $2 h_\text{geo}$ as one would measure with a ruler. Due to fringe fields, $h_a>h_\text{geo}$. We assume the magnetic height of the air gap is known.

For the typical large permeabilities of the yoke materials, the metal on each side of the air gap is a magnetic equipotential surface. Hence, the magneto motive force over the air gap given by $\int _{r_i}^{r_o}H(r)\hbox {d}r$ with $r_i$ and $r_o$ denoting the inner and outer radius of the air gap, does not change when the coil is introduced and is independent of the vertical position *z* where the integration is performed. The magnetic field *H* is the magnetic flux divided by the permeability, $H=B/(\mu _0(1+\chi ))$ and is a function of radius and height $H(r,z)=H(z)r_c/r$, where we have used the fact that the field drops off as 1/*r* and *H*(*z*) is the field at the mean coil radius $r_c$.

Two paths of integration through the coil at $z=z_c$ and in the empty air gap ($z=z_\chi$) are shown in Fig. 2f. Integration along these paths yield

15 $$\begin{aligned} \int _{r_i}^{r_o}\frac{H(z_\chi ) r_c}{r}\hbox {d}r= & {} \int _{r_i}^{r_o}H(r,z_c)\hbox {d}r \nonumber \\ \int _{r_i}^{r_o}\frac{B_\chi r_c}{\mu _0r}\hbox {d}r= & {} \int _{r_i}^{r_l}\frac{B_cr_c}{\mu _0r}\hbox {d}r+\int _{r_l}^{r_r}\frac{B_c r_c}{\mu _0r(1+\chi )}\hbox {d}r+\int _{r_r}^{r_o}\frac{B_c r_c}{\mu _0r}\hbox {d}r\nonumber \\ \int _{r_i}^{r_o}\frac{B_\chi }{r}\hbox {d}r\approx & {} \int _{r_i}^{r_o}\frac{B_c}{r}\hbox {d}r-\int _{r_l}^{r_r}\frac{\chi B_c }{r}\hbox {d}r, \end{aligned}$$

where $r_l=r_c-w_c/2$ and $r_r=r_c+w_c/2$ denote the inner(left) and outer(right) edge of the coil. The quantities *B*(*r*) and *H*(*r*) as a function of *r* for both integration paths are shown in Fig. 2g,h, respectively. Integrating the terms, approximating the resulting logarithms in a Taylor series of first order and combining the result with Eq. (14) gives

16 $$\begin{aligned} B_0=B_\chi \left( 1+\chi \frac{w_c h_c}{w_a h_a}\frac{r_a}{r_c} \right) . \end{aligned}$$

With Eq. (13) the relative change that the magnetic material has in the velocity mode can be stated as

17 $$\begin{aligned} \frac{\Delta U_\chi }{U}=\frac{B_\chi }{B_0}-1\approx -\chi \frac{A_c}{A_a}\frac{r_a}{r_c}. \end{aligned}$$

As before, $A_c=2h_cr_c$ and $A_a=2h_ar_a$ denote the cross-sectional areas of the coil and the air gap, respectively.

Equation (17) shows the relative change of the induced voltage in the velocity mode is identical to the relative change in force mode, see Eq. (12). The robustness of Kibble’s reciprocity to deviations from the ideal experimental setup without magnetic materials, are caused by a strong symmetry in the underlying physics. Without that robustness the Kibble balance would not be the success that it has been in metrology. The relative differences of the measured force and in voltage from the corresponding ideal theoretical values in the absence of weakly magnetic materials are given by

18 $$\begin{aligned} \frac{U_{\mathrm {real}} - U_{\mathrm {ideal}}}{U_{\mathrm {ideal}}} =\frac{F_{\mathrm {real}} - F_{\mathrm {ideal}}}{F_{\mathrm {ideal}}} \approx -\chi \frac{A_c}{A_a}\frac{r_a}{r_c}. \end{aligned}$$

Since $r_a \approx r_c$, the relative effect is proportional to the magnetic susceptibility and the cross-sectional filling ratio of the air gap. The latter denotes how much of the cross-sectional area of the air gap is taken up by the coil. With typical values, $\chi =-10^{-5}$ and $A_c/A_a=0.1$, the relative difference between the real and ideal numbers is $1\times 10^{-6}$. In conclusion, the diamagnetic force with a relative magnitude of $1\times 10^{-6}$ about 100 times larger than the reported relative uncertainties exists. But the results of the Kibble balance experiments are not affected by it, because the same relative bias will be introduced in the velocity mode. In the combination of the measurement results from force and velocity mode, the effect cancels perfectly.

The derivation above has been made using ideal geometries to show the powerful and simple idea. But, the theory holds for more complex and realistic field situations, as is discussed in the “Methods” section.

### Numerical verification

Numerical verification of a relative force change that is as small as $10^{-6}$ is impossible. Since engineering tasks are rarely concerned with effects that small in size, commercial finite element programs are not optimized for the precise prediction of these small effects. At this order of magnitude their results cannot be trusted. To overcome their limitations and to be able to use commercial finite element analysis (FEA) software, we invented a new technique that we name differential FEA (dFEA). While more information on dFEA can be found in the supplemental information, the following paragraphs explain the general idea. All effects discussed here are proportional to the magnetic susceptibility $\chi$ and it can be used as a parameter to verify the result. Setting $\chi$ to a large value amplifies the relative change in force and voltage. With $\chi \approx 1\times 10^{-2}$, relative effects of $1\times 10^{3}$ are achieved. Although the theory is only weakly dependent on geometry and independent of the size of the magnetic field, typical values are used. A magnetic flux density of $B_0=0.54\,$T was chosen, and it requires $+/-11.6$ A in a single turn to produce half the weight of a kg standard in positive/negative vertical direction. Ansoft a commercial FEA software was used to calculate the force produced on a coil consisting of a single turn. For all calculations shown below an adaptive mesh strategy and a nonlinear solver were used. The calculations were performed with five different values for the magnetic susceptibility of the coil wire ranging from − 0.01 to 0.01. The precise result of the calculation depends on how the geometry is meshed by the FEA software. To avoid any bias in this investigation, the mesh is only calculated once and fixed for all subsequent calculations. For each $\chi$ value, the force on the coil without current is calculated. Then the forces for positive and negative currents are calculated. From both the null result is subtracted. This differential approach suppresses systematic errors in the calculation due to meshing and rounding of the small effects.

**Figure 3:**
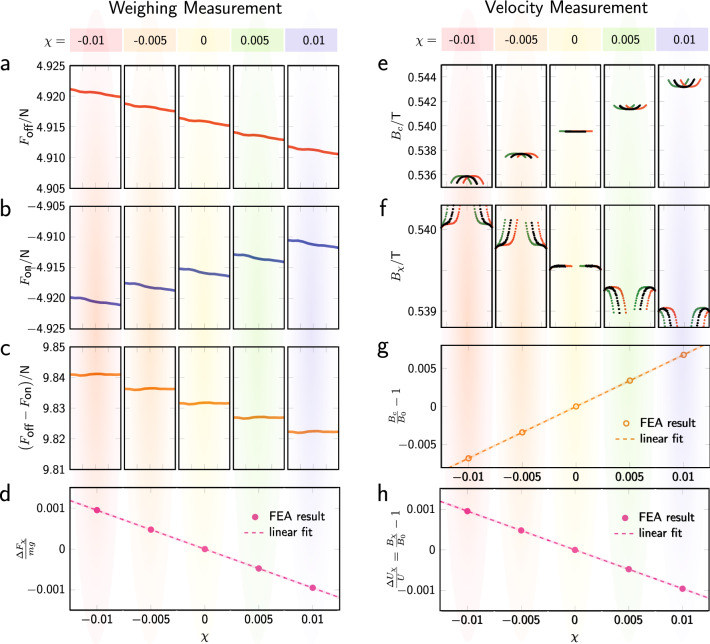
Results of the magnetization effect in weighing and velocity measurements. (**a**)–(**c**) Results of magnetic force as a function of coil position − 10 mm $\le z\le$ 10 mm for five different magnetic susceptibilities. The current in the coil was equivalent to one turn with $I_\text{off}$= − $I_\text{on}$=11.6 A. (**d**) The relative change in force difference as a function of $\chi$. Note that *mg* is defined at force difference at $\chi =0$. (**e**) The $B_c$ distribution at $z=\pm 6$ mm and $z=0$ mm with different $\chi$ values. No current is assigned in the calculation. (**f**) shows the $B_\chi$ field distribution. (**g**) presents the relative magnetic field change of $B_{c}$ and (**h**) shows the change of $B_{\chi }$.

The calculated force differences with positive (mass-off) and negative (mass-on) currents are shown in Fig. 3a,b, respectively. In each subplot, the force is given as a function of coil position *z* with $-10\,\hbox {mm}\,\le z\le \,{10}\,\hbox {mm}$ for the five $\chi$ values. The clearly visible slope in each subplot is caused by the reluctance force, in agreement with the theoretical model discussed in Ref.^48^. The slope is the same for both current directions and independent of $\chi$. Hence, in the force difference, shown in Fig. 3c, the slope vanishes and the difference is nearly independent to *z*, exactly as described in Ref.^48^. Neglecting the slopes, the observed values of $F(I_{\mathrm{off}})$ and $F(I_{\mathrm{on}})$ at a given point, for example $z=0$, change with $\chi$. The change of the force difference relative to the weight of a 1 kg mass at $z=0$ is shown in Fig. 3d.

The size of the force (difference) depends on $\chi$. Diamagnetic materials ($\chi <0$) yield larger absolute values for the forces for both current directions. Unlike the slopes, this effect does not cancel by subtracting mass-on from the mass-off measurement. The effect is clearly visible in Fig. 3c where the force differences are plotted.

A linear dependence of the force differences on $\chi$ is observed, see the dashed line in Fig. 3d. The slope of the line can be obtained by a numerical regression to the calculation results, and by using the regression coefficients the effect can be scaled down to small $\chi$ values whose results would otherwise be in the rounding error of the numerical analysis. For $\chi =-1\times 10^{-5}$ a relative change of $9.50\times 10^{-7}$ is obtained, in very good agreement to the theoretically obtained result of $1\times 10^{-6}$. The numerical results confirm the theoretical analysis, as well as the existence of the diamagnetic force.

The same FEA calculation can be used to estimate the effect in the velocity mode. Here, we calculate the magnetic flux density in the air gap for a coil that does not carry any current for the five susceptibilities discussed above. As in the text above, two symbols are used to describe the flux density in the air gap in the presence of magnetic material. At regions that the coil occupies we use $B_c$ and all other regions $B_\chi$.

Figure 3e,f show the magnetic flux densities $B_c$ and $B_\chi$ as a function of *z*, with the coil at three different positions $z_c$, $z_c={-6}\,\hbox {mm}$ in green, $z_c=0\,\hbox {mm}$ in black, and $z_c=6\,\hbox {mm}$ in red. For a given $\chi$, one of the two quantities $B_c$ and $B_\chi$ is larger and the other smaller than $B_0$, which can be seen in the two middle panels with $\chi =0$. The curves of $B_\chi$ show a transient step at the border close to $B_c$. This is an artifact of the FEA calculation which cannot reproduce the perfect step function in *B* that would be present at the boundary in the real world, see Fig. 2b. We believe that the transient has no influence on the conclusion, especially since its integral evaluates to zero. For any $\chi \ne 0$, $B_\chi -B_0$ is about a seventh of $B_0-B_c$, and, hence, according to Eq. (14), the effective gap height is about eight times the coil height, such that $h_c/(h_a-h_c)=1/7$.

To consolidate this assertion, a summary of the relative difference of $B_c$ and $B_\chi$ with respect $B_0$ are shown in Fig. 3g,h. Both figures are plotted for $z_c=0$. The former shows $B_c(0)$ the latter $B_\chi$ near the end of the gap, both are relative to $B_0$ at the same locations. Similar to the results in the force mode, the results are linear with respect to the chosen magnetic susceptibility and a regression to the calculation results is performed. From the regression coefficients, $B_{\chi }/B_0-1$ can be calculated for small $\chi$, a result that would be unobtainable directly from finite element analysis. For $\chi =-1\times 10^{-5}$, $B_{\chi }/B_0-1$ is $9.51\times 10^{-7}$. For comparison, the relative effect in force mode for the same $\chi$ was $9.50\times 10^{-7}$. The calculated relative effects in force mode and velocity mode agree remarkably well (the difference of $1\times 10^{-9}$ is negligible compared to the numerical uncertainty of the FEA calculation).

The summary of this section is given in the last row of Fig. 3. The left graph shows the relative bias that is incurred in force mode as a function of the magnetic susceptibility of a weakly magnetic coil. The right graph shows the relative bias incurred in velocity mode as a function of the same $\chi$. The results are identical, the relative biases depend linearly on $\chi$. For the model discussed here the slope of the line is approximately $-1/10$, which corresponds to the fraction of the cross sectional area of the air gap that is filled by the coil. So, a Kibble balance with this geometry and a weakly magnetic coil would produce values for both modes that differ by $-\chi /10$ compared to the same balance that has a completely nonmagnetic coil. However, when the results from the force and velocity mode are combined according to Eq. (3), the relative biases cancel each other and the mass measured by the Kibble balance with a weakly magnetic coil is identical to the mass measured by a Kibble with a non-magnetic coil.

## Discussion

The work that led to this article accomplished four tasks.

1. We have shown that the diamagnetic effect in force mode exists and its relative magnitude can be as large as $1\times 10^{-6}$.
2. We have discovered a corresponding effect in velocity mode that completely cancels out the effect of the diamagnetic force in the Kibble balance experiment. Such an effect has never been described before in the literature.
3. We have developed a new technique to calculate very small magnetic effects caused by weakly magnetic materials using finite element analysis.
4. By using the newly developed technique we could verify the existence of (1) and (2) and show that they have the same relative size within the numerical uncertainty.

Below we summarize the most important points for these accomplishments.

The force described by the diamagnetic effect exists and it is large ($\approx 1\times 10^{-6}$) compared to the relative uncertainties that Kibble balances report ($\approx 1\times 10^{-8}$). A Kibble balance requires a coil immersed in a magnetic field. Often the magnet wire is made from copper that is weakly diamagnetic with $\chi =-1\times 10^{-5}$. Without current a diamagnetic force on the coil wire exists, but it is a constant force comparable to the weight of the coil and will not impact the result. What is understood as the diamagnetic effect is caused by the current in the coil during the weighing measurement. This current generates an additional magnetic field which interacts with the magnet system in what is known as back-action. Due to the back-action, the diamagnetic force is no longer constant, but proportional to the current in the coil, and, hence, it no longer cancels and provides a systematic bias in the weighing measurement of the Kibble balance experiment. The relative size of this effect can be written very compact, see Eq. (12). If the coil and the air gap have the same radius the effect is proportional to the magnetic susceptibility and the ratio of the cross-sectional areas of the coil and the air gap.

Unbeknownst to the scientist and engineers working with Kibble balances, there is also an effect in velocity that arises when a weakly magnetic material is added into the gap. Introducing such a material in the gap changes the magnetic flux density and hence the result that is obtained in the velocity mode. Adding, for example, a diamagnetic coil in the gap reduces the magnetic flux density where the coil is and increases the magnetic flux density in the remainder of the gap. This is a consequence of the changed reluctance of part of the gap. Where the coil is the magnetic reluctance is larger leading to a smaller amount of flux. However, since the flux through the total air gap remains approximately constant, the flux at the remainder of the gap increases. The increased flux causes a larger induced voltage when the coil is moved through the gap compared to the situation where the coil is non-magnetic. As shown in Eq. (17), the relative change in voltage evaluates to the same expression as for the diamagnetic force. Hence the bias introduced in the weighing mode is cancelled by an equal bias in velocity mode. Thus, the paradox of the diamagnetic force on the Kibble coil is resolved.

To prove the existence of the effect of weakly magnetic materials in force and velocity mode we have developed a new technique that we call differential finite element analysis (dFEA). Calculating small forces or field changes caused by the introduction of materials whose susceptibility is of order $1\times 10^{-5}$ is impossible. The numerical uncertainties are much larger than the relative effects one desires to calculate. In differential FEA, the susceptibility of the material to be investigated is a parameter and the model is calculated with several different large susceptibilities. Values of $\chi \le 1\times 10^{-2}$ were used up to a thousand times larger than the susceptibility of the coil in the physical experiment. For differential FEA to work, it is important to keep the same mesh for all calculations. From each calculation result, a null-result that was obtained by setting $\chi$ equal to zero is subtracted. In the end, the quantity of interest is plotted as a function of the used $\chi$ and a smooth function is fitted to the result. The fitted parameters of the function can be used to calculate the effect for small $\chi$ that the physical system has. For the cases discussed here, both effects scaled linearly with $\chi$ making the scaling simple.

We used differential FEA to calculate the effect that the introduction of a weakly magnetic material has on measurements in force and velocity mode. We find the calculated result in agreement with a simplified analytical model that we have developed in the preceding sections. The relative sizes of the effect are of order $1\times 10^{-6}$ and would render Kibble balances useless. The effect has the same magnitude and sign in both modes and will cancel in the combined result. We believe that this is an additional, to date not recognized symmetry of the Kibble balance that allows it to work in the presence of linear magnetic materials. The result of the differential FEA shows that the biases in force and velocity agree within a difference of $1\times 10^{-3}$, limiting the upper bound for the relative bias of the combined measurement to $1\times 10^{-9}$.

The paradox of the diamagnetic force in Kibble balances has been solved. The ongoing discussions in the Kibble balance community are brought to a satisfying end. The reciprocity of Kibble’s equation works perfectly in the presence of linear magnetic materials.

## Methods

### Self-inductance *L*(*z*) of a coil in a symmetrical yoke

Let $w_a$, $2h_\text{geo}$, and $r_a$ be the geometrically measured width, height, and mean radius of the air gap. Neglecting the fringe fields at the end, the air gap has a magnetic reluctance of $R_\text{ideal}={w_a}/{(4\pi \mu _0 r_ah_\text{geo})}$. The relative correction necessary to account for the fringe fields scales with gap’s aspect ratio $w_a/(2h_\text{geo})$^49^. The reluctances of the leakage paths at both ends of the gap are parallel to $R_a$, lowering the total reluctance of the system. Writing the reduction factor as $1/\gamma$ with $\gamma >1$, yields $R_a={w_a}/{(4\pi \mu _0 r_ah_\text{geo}\gamma )}$, which can be interpreted as the reluctance of an ideal air gap of the same dimension, but a magnetic height, $2h_a$, that differs from the geometric one according to $h_\text{a} = \gamma h_\text{geo}$. In our idealized gap, the magnetic flux is purely horizontal. Neglecting the vertical flux, makes the analysis simpler without altering the conclusion.

Now, let’s investigate the flux that is produced by a coil at position $z_c$ with *N* turns carrying a current *I*. The flux $\phi _0$, generated by the magneto motive force of the coil, has to traverse the air gap above and below the coil. Hence,

19 $$\begin{aligned} \phi _0=\frac{NI}{(R_u+R_l)},~~\hbox {where}~~R_u=\frac{w_a}{2\pi \mu _0 r_a(h_a-z_c)},~~R_l=\frac{w_a}{2\pi \mu _0 r_a(h_a+z_c)}, \end{aligned}$$

where $R_{u/l}$ denote the magnetic reluctance of the partial gap that is above/below the coil. Using the definition of the self-inductance $L={N\phi _0}/{I}$ and expanding the fractions to second order in $z_c$ yields

20 $$\begin{aligned} L=2\pi \mu _0 N^2 r_a\left( \frac{ h_a}{2w_a }-\frac{z_c^2}{2w_a h_a}\right) . \end{aligned}$$

which appears in the text as Eq. (11). Experimentally, the inductance of the coil can be measured as a function of $z_c$. By fitting $L=L_0-kz_c^2$ to the data, the magnetic height of the air gap can be determined from *k* as

21 $$\begin{aligned} 2h_a=\frac{2\pi \mu _0N^2 r_a }{w_a k}. \end{aligned}$$

For the magnet employed by the BIPM Kibble balance, $k\approx 550\,\hbox {H}\hbox { m}^{-1}$, see Ref.^46^. Using the reported technical data of that magnet system, $w_a$=13 mm, $N=1057$ and $r_a=125$ mm, a magnetic height of $2h_a=155\hbox { mm}$ is obtained. A comparison to the measured, geometric height, $2h_\text{geo}=82$ mm shows that $\gamma =1.89$.

The magnetic height can also be deduced from a finite element analysis (FEA), employing for example Eq. (14). The value for $2h_a$ obtained by the measurement of *L*(*z*) agrees with the one obtained by FEA within a few percent. The difference is due to the fact that magnet’s top cover was missing in the experiment.

### General equation of a moving cylindrical segment with finite $\chi$

Here, we investigate the effect cause by any weakly magnetic part that is co-moving with the coil. Such parts are abundant in any Kibble balance experiments and include the coil former, the supporting frame, optical elements, and fasteners. Without loss of generality, we investigate a cylindrical part with a rectangular cross section identified by the subscript $_i$, its height, width, mean radial location, and magnetic susceptibility are denoted by $2h_i$, $w_i$, $r_i$, and $\chi _i$. The symmetry axis of the part coincides with the symmetry axis of the magnet.

Starting with Eq. (9), the diamagnetic force in weighing mode on the segment is

22 $$\begin{aligned} \Delta F_{\chi ,i} = \frac{2 \chi _i V_i}{\mu _0}I \partial _z\left( B_{v,i}^2 \alpha \right) , \end{aligned}$$

where $V_i=2\pi r_i(2h_iw_i)$ is the volume of the cylindrical segment and $B_{v,i}$ the magnetic flux density at the segment position without current in the coil. The flux produced by the permanent magnet and the coil are both horizontal, and, hence, the flux density is proportional to 1/*r*. Consequently, at the mean radial position of segment *i*, the flux is $B_{v,i}=({r_c}/{r_i})B_v$, yielding

23 $$\begin{aligned} \frac{\Delta F_{\chi ,i}}{\Delta F_{\chi }} = \frac{\chi _i}{\chi } \frac{V_i}{V}\left( \frac{B_{v,i}}{B_v}\right) ^2 = \frac{\chi _i}{\chi } \frac{r_c}{r_i}. \end{aligned}$$

Using the expression for the diamagnetic effect on the coil (12) together with (23), produces a compact expression for the relative diamagnetic force produced by the segment. It is

24 $$\begin{aligned} \frac{\Delta F_{\chi ,i}}{mg}=-\chi _i \frac{A_i}{A_a} \frac{r_a}{r_i}, \end{aligned}$$

where $A_i=2h_iw_i$ the sectional area of segment *i*, and $A_a$ the cross sectional area of the air gap defined above.

Most importantly and similarly to Eq. (12), the relative diamagnetic force on segment *i* is independent of the coil current and the magnetic field $B_v$, and is determined only by the material property ($\chi _i$) and geometrical ratios ($A_i/A_a$ and $r_a/r_i$).

Next, the influence of the weakly magnetic segment *i* on the measured value of *Bl* in velocity phase is investigated. Assuming a magnet system with perfect up-down symmetry, as shown in Fig. 1a, we define the following three surfaces at $r=r_c$: $\mathscr {A}$ ($r\le r_c$, $z=h_a$) and $\mathscr {B}$ ($r\le r_c$, $z=-h_a$) present the horizontal surfaces respectively at the upper and lower gap ends. $\mathscr {C}$ ($r\le r_c$, $z=z_c$) is the coil surface. In perfect symmetry, the magnetic flux $\phi _\mathscr {A}$ penetrating surface $\mathscr {A}$, equals the flux $\phi _\mathscr {B}$ through surface $\mathscr {B}$. An asymmetry can be taken account by introducing a flux difference $\Delta \phi _0$ such that

25 $$\begin{aligned} \phi _\mathscr {A}=\phi _\mathscr {B}+\Delta \phi _0, \end{aligned}$$

By using an electrical circuit model following Ohm’s law of magnetism as shown in Fig. 1a, $\phi _\mathscr {A}$ and $\phi _\mathscr {B}$ are determined as

26 $$\begin{aligned} \phi _\mathscr {A}=\frac{E_1}{(R_m+R_a)}\;\;\hbox {and}\;\;\phi _\mathscr {B}=\frac{E_2}{(R_m+R_a)}. \end{aligned}$$

A magnetic segment co-moving with the coil, does not contribute to the air gap reluctance $R_a$, and, hence, $R_a$ does not depend on the vertical position of the segment. Since $\phi _\mathscr {A}$, $\phi _\mathscr {B}$ are constant for a given magnet system, the flux difference $\Delta \phi _0$ must also be independent of the coil position $z_c$.

That flux that goes through surface $\mathscr {A}$ will then either go through the coil or through the part of the air gap that is above the coil $\phi _\mathscr {U}$, $\phi _\mathscr {A}=\phi _\mathscr {C}+\phi _\mathscr {U}$. Similarly, all the flux penetrating $\mathscr {B}$ flows through the part of the air gap that is below the air gap or through the coil, $\phi _\mathscr {B}=-\phi _\mathscr {C}+\phi _\mathscr {L}$. The negative sign before the coil flux indicated the direction of the flux relative to the normal vector of the coil. It is reverse for the flux $\phi _\mathscr {B}$.

The fluxes $\phi _\mathscr {U}$ and $\phi _\mathscr {L}$ can be written as a product of the surface area and the magnetic field at the radius $r_i$ under the assumption that the field is mostly independent of *z*. Hence,

27 $$\begin{aligned} {\left\{ \begin{array}{ll} \phi _\mathscr {U}(\chi _i=0)=2\pi r_i (h_a-z_c)\,B_{0,i}, \\ \phi _\mathscr {L}(\chi _i=0)=2\pi r_i(h_a+z_c)\,B_{0,i} \end{array}\right. } \end{aligned}$$

the flux through the coil can now be obtained as

28 $$\begin{aligned} \phi _\mathscr {C}(\chi _i=0)=2\pi r_iB_{0,i}z_c+\frac{\Delta \phi _0}{2}. \end{aligned}$$

To evaluate the induced voltage only the component that depends on *z* is relevant and we obtain

29 $$\begin{aligned} U(\chi _i=0)=2\pi r_iNB_{0,i}v_{z}. \end{aligned}$$

Taking again advantage of the 1/*r* dependence of the magnetic flux density, $B_{0,i}=r_c/r_i B_{0}$. For $\chi =0$, the voltage-velocity ratio is the $2\pi r_cNB_{0}=B_0l$ in agreement with the conventional theory of the Kibble balance.

For $\chi _i\ne 0$, the magnetic field distribution along the vertical direction at $r_i$ is given by discrete two values with sharp steps between them. In the coil region (from $z_i-h_i$ to $z_i+h_i$), the magnetic flux density is $B_{c,i}$ and the magnetic flux density of the rest air gap is $B_{\chi ,i}$. In this case $\phi _U$ and $\phi _L$ are written as

30 $$\begin{aligned} {\left\{ \begin{array}{ll} \phi _\mathscr {U}(\chi _i)=2\pi r_i\left[ B_{c,i}\eta _i 2h_i+B_{\chi ,i}\left( h_a-z_c-\eta 2h_i\right) \right] ,\\ \phi _\mathscr {L}(\chi _i)=2\pi r_i\left\{ B_{c,i} (1-\eta _i)2h_i+B_{\chi ,i}\left[ h_a+z_c-(1-\eta _i) 2h_i\right] \right\} , \end{array}\right. } \end{aligned}$$

where $\eta _i$ defines the height fraction of the segment that is above the coil $z_c$. For example, when segment *i* is fully above the coil, $\eta _i=1$. If segment *i* is coincident with the coil (like the coil itself), then $\eta _i=0.5$. Note since the segment is co-moving with the coil, $\eta _i$ does not change with $z_c$. Through Eq. (30) and known magnetic flux relations, the magnetic flux through the coil is solved as

31 $$\begin{aligned} \phi _\mathscr {C}(\chi _i)= & {} 2\pi r_i\left[ B_{\chi ,i}\left( z_c+(2\eta _i-1)h_i\right) -B_{c,i}(2\eta _i-1)h_i\right] +\frac{\Delta \phi _0}{2}. \end{aligned}$$

Dismissing all factors that are independent of $z_c$, the induced voltages simplifies to

32 $$\begin{aligned} U(\chi _i)=\frac{N\partial \phi _C(\chi _i)}{\partial t}=2\pi r_iNB_{\chi ,i}v_{z}. \end{aligned}$$

Comparing Eqs. (29) to (32), the relative change in the induced voltage cause by segment *i* is

33 $$\begin{aligned} \frac{\Delta U_{\chi ,i}}{U}=\frac{B_{\chi ,i}}{B_{0,i}}-1. \end{aligned}$$

This result is the equivalent expression as given in Eq. (13). The result of $B_\chi /B_0-1$ remains valid and the relative change in the induced voltage is

34 $$\begin{aligned} \frac{\Delta U_{\chi ,i}}{U}=\frac{B_{\chi ,i}}{B_{0,i}}-1=-\chi _i \frac{A_i}{A_a} \frac{r_a}{r_i}. \end{aligned}$$

The relative effects in force and velocity mode caused by the introduction of a cylindrical segment *i* with finite susceptibility are identical, compare Eqs. (24) to (34).

The relative changes of the induced voltage and the extraneous force produced by a single segment *i* depend on the ratios $A_i/A_a$ and $r_i/r_a$. Hence, the scaling can be checked by comparing the relative effects produced by two different segments *i* and *j* with the same magnetic susceptibility. The ratio of the relative effects must scale like $(A_i/r_i)/(A_j/r_j)$. The calculation was performed for both segments for both modes, velocity mode and force mode. Again, differential finite element analysis as described in the main text was used to interpolate the effects to $\chi _j=\chi _i=-1\times 10^{-5}$ by using calculations that used susceptibilities ranging from − 0.01 to 0.01.

As segment *i*, we use the coil which has been already shown in the main text and, for convenience, we reiterate the numbers here. The coils has a cross-sectional area of $A_i={200}\,\hbox {mm}^2$ and a mean radius of $r_i={125}\,\hbox {mm}$. It produces a relative effect of $9.50\times 10^{-7}$.

For the segment *j* we chose an area of $A_j={50}\,\hbox {mm}^2$. It is located at $r_j={122.5}\,\hbox {mm}$. To also check if the calculation works for objects that are offset from the vertical coordinates of the coil, we placed segment $j\,17.5\,\hbox {mm}$ above the coil. The relative effect for segment *j* is the same for force and velocity mode and it is $2.43\times 10^{-7}$.

The ratio of the two relative effects is 3.92 and the ratio of the corresponding geometrical factors $(A_i r_j) / (A_j r_i)=3.92$. The agreement between the geometrical ratios and the calculated effect validate Eqs. (24) and (34).

## Supplementary information

Supplementary Information.
