# Supplementary material for: Resolution of the paradox of the diamagnetic effect on the Kibble coil
Source: Sci Rep. 2021 Jan 13;11:1048. doi: 10.1038/s41598-020-80173-9 (PMC7806723; doi:10.1038/s41598-020-80173-9)
Supplement: Supplementary file 1 — Supplementary Information. [file 41598_2020_80173_MOESM1_ESM.pdf]

Supplementary Information for 'Resolution of the paradox of the diamagnetic effect on the Kibble coil'

Shisong Li[1*], Stephan Schlamminger[2+], Rafael Marangoni[2], Qing Wang[1], Darine Haddad[2], Frank Seifert[2], Leon Chao[2], David Newell[2], Wei Zhao[3]

1. Department of Engineering, Durham University, Durham DH1 3LE, United Kingdom
2. National Institute of Standards and Technology, Gaithersburg 20899, United States
3. Department of Electrical Engineering, Tsinghua University, Beijing 100084, China

*Email: shisong.li@durham.ac.uk

+Email: stephan.schlamminger@nist.gov

I. THE MAGNETIC FIELD PRODUCED BY A COIL IN FREE SPACE AND INSIDE A YOKE

In this section, we compare the magnetic field of a current-carrying coil in free space and in a Kibble balance magnet. Figure 1(a) shows the effect that the current in the coil has on the measured Bl in force and velocity mode. We present a summary of the finding that have been described in [1]. Plotted on the vertical axis is the difference of Bl measured in different scenarios from a velocity phase measurement $(U/v)_0 = B_0 l$ where the coil is moving through the magnetic field without any current. The two profiles $(F/I)_{\mathrm{off}}$ and $(F/I)_{\mathrm{on}}$ are used in most Kibble balances for weighing – usually the two measurements in weighing phase, mass-on and mass-off, are carried out with equal and opposite currents, $I_{\mathrm{off}} = -I_{\mathrm{on}}$. The profiles $(U/v)_{\mathrm{off}}$ and $(U/v)_{\mathrm{on}}$ are profiles determined in the velocity phase, respectively with plus and minus currents. The bifilar coil used in the BIPM balance allows current to be present during the velocity mode. For conventional two-mode Kibble balances, $(U/v)_0$ is used, while $(U/v)_{\mathrm{off}}$ and $(U/v)_{\mathrm{on}}$ are for one-mode measurement schemes, e.g. [2–4].

The data in figure 1(a) shows that the change in Bl is a linear in coil position z, as well as in coil current I in the weighing measurement. As discussed in the article, the field gradient $\partial_z B$ produces a diamagnetic force bias that can not removed by mass-on and mass-off measurements. The interesting conundrum is, can the magnet field gradient that is produced by the coil itself produce a force on itself.

To clarify the paradox we study two scenarios: 1) the coil in free space and 2) the coil inside an air gap surrounded by iron yokes as it would in a Kibble balance . Figure 1(b) and (c) show the magnetic flux density distribution along z for different weighing positions and both current directions. The results shown in the figure are obtained by finite element analysis based on the BIPM magnet system [5] with an average field in the air gap of $B_0 = 0.45\,\mathrm{T}$. To obtain the free-space calculation, the yoke relative permeability is simply set to one. Using the same geometry and mesh, results in a trustworthy comparison of both scenarios.

Figure 1(b) clearly shows that the magnetic field distribution is independent of the vertical position for the coil in free space. The size and shapes of the green, orange and violet lines are identical only horizontally displaced by 10 mm from each other. Hence, energetically it does not matter where the coil sits. There is no minimum and hence no force on the coil. Although magnetic gradients exist on the curve $B(z)$ of a free space system, any force related to these gradients is internal (the coil gets compressed), which cannot be seen by the weighing unit

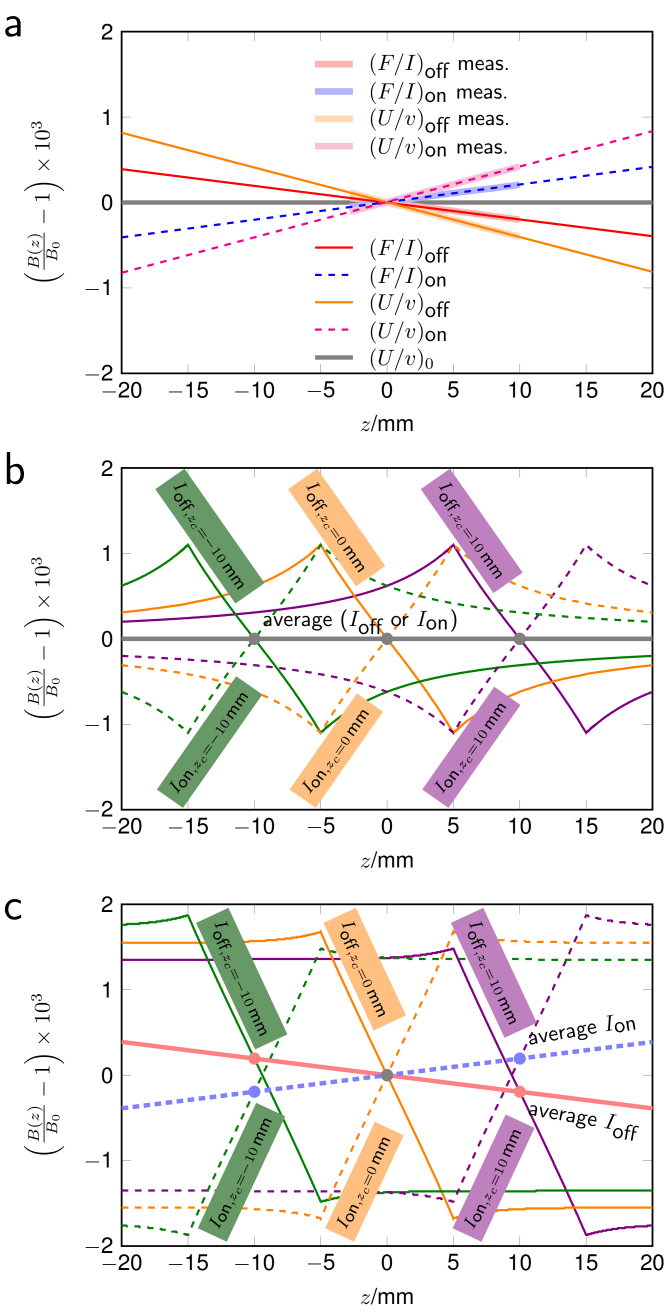

FIG. 1. (a) The magnetic field change due to the coil current. The middle thick curves are obtained by experimental measurement in the BIPM system[1] and the thin lines are linear extrapolations from -20 mm to 20 mm. (b) presents the relative B field distribution when the yoke permeability is set to μ_0 and (c) shows the magnetic field change with soft normal yoke. Note the reference field, $B_0 = 0.45\,\text{T}$, is used for both cases (b) and (c).

(balance or mass compactor).

For the coil in he yoke, shown in figure 1(c), the situation is different. The additional fields are only symmetric for $z = 0\,\text{mm}$. At all other positions ($z \neq 0\,\text{mm}$) the symmetry is broken. To one side of the coil the magnetic flux density is higher to the other it is lower. As was shown above, it is not the local gradient of the field that provides information on the force. The volume average over the coil must be considered. The volume average, shown as the blue and red line in the figure, is no longer independent of z as it was in the free space scenario.

The external force on the coil depends on the average field. The experimental observation, shown in figure 1(a) agrees well with the FEA calculation presented in 1(c).

The comparison of average fields produced by a current-carrying coil in free space and in a yoke shows that in the latter the broken symmetry will lead to an energy redistribution and hence a magnetic field change. A physical picture can be provided as follows: In the yoke B changes because the coil current magnetizes the yoke, and the magnetized yoke produces an additional magnetic field at the coil position.

The diamagnetic force arises from $F_\chi = \frac{\chi V}{\mu_0} B \partial B_z$, where the last factor is the derivative of the volume average field with respect to the coil position. As shown in figure 1(c), the slopes of the red and blue lines and, hence the gradient ∂B_z is constant within reasonable ranges of z. Accordingly, the diamagnetic force is constant as a function of z, but changes direction when the current is reversed. Because of the latter fact, the diamagnetic force does not cancel in the Kibble balance experiment and can lead to a systematic bias.

In conclusion, the statement that ‘a current-carrying coil can not produce a measurable force on itself’ holds for free space system, but not necessarily for a coil inside a yoke, unless the yoke moves with the coil. We note that this finding does not contradict Newton’s third law as there is an equal and opposite force on the yoke.

II. DIFFERENTIAL FEA (DFEA) – A METHOD TO CALCULATE SMALL EFFECTS

The diamagnetic effects described in the main article are very small. In force mode, an additional bias of 10 µN on top of 10 N is produced. It would be impossible with finite element analysis to detect, let alone to calculate with any uncertainty, this additional bias. We first developed the analytical equations of the diamagnetic effect in force and velocity modes. However, we wanted to verify our analytic results with numerical calculations. In searching for ways to do this, we invented differential FEA (dFEA). With differential FEA, it was possible to calculate the size of the effect with a relative uncertainty of about 0.1%, corresponding to f 10 nN. We could further show that the diamagnetic effect produces the same bias in force and velocity mode within that uncertainty.

Conventional finite element analysis, FEA, is a powerful technique to solve a variety of engineering problems that would be difficult or impossible to solve analytically. The basic idea of FEA is to divide the domain under investigation into small elements, and then to approximate the solution of the problem by a linear combination of calculations in each element. In FEA simulations, small errors are inevitable. Either discretization or numerical errors cause these problems. The computer simulates a discrete system, while the real system in nature is continuous. Numerical errors can occur, for example, by rounding when two large numbers are subtracted or by ill-conditioned matrices. In our experience, problems in magnetostatic can be calculated with relative errors of a few parts in 10^3 on a standard PC in reasonable time. The effects discussed in our work are of the order 1×10^{-6}. Hence, they are three orders of magnitude smaller than what we consider reliable results obtainable by FEA.

With dFEA, small effects and their uncertainty can be calculated using commercial FEA packages without exponentially extending the computation time. The technique takes about five times longer than a single calculation because, as the reader shall see below, the same model has to be calculated about five times with different χ. The idea is simple. As mentioned above, the

uncertainties of the calculations are 1000 times larger than the effect, so if one can increase the effect by a factor of 1000, the effect could be detected. In our case, where the effect scales with the susceptibility, one only needs to multiply the susceptibility of the part that is investigated by a factor of 1000 in question by a factor of 1000 to achieve that amplification. So, if χ_{nom} is the susceptibility of the part in question, here the coil, $\chi_{\mathrm{exag}} \approx 1000 \times \chi_{\mathrm{nom}}$ is used in the finite element calculation. It is safe to change the susceptibility because it occurs as a linear parameter, and the underlying physics does not change. In the end, all one has to do is scale the measured effect, say the force, by $\chi_{\mathrm{nom}}/\chi_{\mathrm{exag}}$ to obtain the size of the previously immeasurable effect.

A successful implementation of dFEA relies on three good practices:

1. It is not sufficient to perform one calculation with χ_{exag} and scale the result by $\chi_{\mathrm{nom}}/\chi_{\mathrm{exag}}$. Usually there are small calculation biases that would be scaled and falsify the result. See, for example, the middle graph of figure 2 , where the force calculated with zero current is not exactly zero. The best practice is to calculate the desired effect for several χ values and then interpolate the size of the effect to the nominal χ. For example, we would like to calculate the diamagnetic effect for $\chi_{\mathrm{nom}} = 1 \times 10^{-5}$. We use five value for χ_{exag}, namely -0.01, -0.005, 0, 0.005, 0.01. We then plot the desired function, for example the force F, as a function of χ_{exag}, subtracting $F(\chi_{\mathrm{exag}} = 0)$. This function is linear in χ_{exag} and the value at χ_{nom} can be obtained from a linear fit to the data.

2. It is of utmost importance to keep all other parameters constant when using dFEA. If additional parameters were changed for the calculation with different χ_{exag}, they would cause a change in the calculation result that would then, falsely, be attributed to being driven by the susceptibility. This point applies, especially to the meshing. The domain should be meshed only once before the various χ_{exag} are assigned to the components.

3. The third practice applies to calculations where current is involved. In this case, the best results are obtained by calculating the system twice, with and without current. The difference between the two results gives the desired effect. Again, other than the current, nothing can change between the calculations, most importantly, not the meshing.

Figure 2 illustrates the importance of the three practices mentioned above. The figure shows the FEA results of the force on the coil at eleven z positions for three different currents, I_{off}, 0, and I_{on}. The forces, as a function of z, show the same trend for all three currents. The trend is caused by numerical biases in the FEA software and affects all three curves in the same, nonphysical, fashion. By subtracting the data obtained with $I = 0$, the numerical biases can be subtracted out. The curves labeled dFEA show relative results.

The curves obtained with dFEA are not only smooth, but they are also physical. They represent the coil inductance force [1], a linear force curve over z. A physical result can be obtained from the original noisy result. The standard deviation of the data calculated with dFEA is 100 times smaller than standard FEA data.

In summary, dFEA allows one to calculate a relative diamagnetic force of 1×10^{-6} with meaningful uncertainties. The method relies on FEA calculations with several exaggerated susceptibilities,

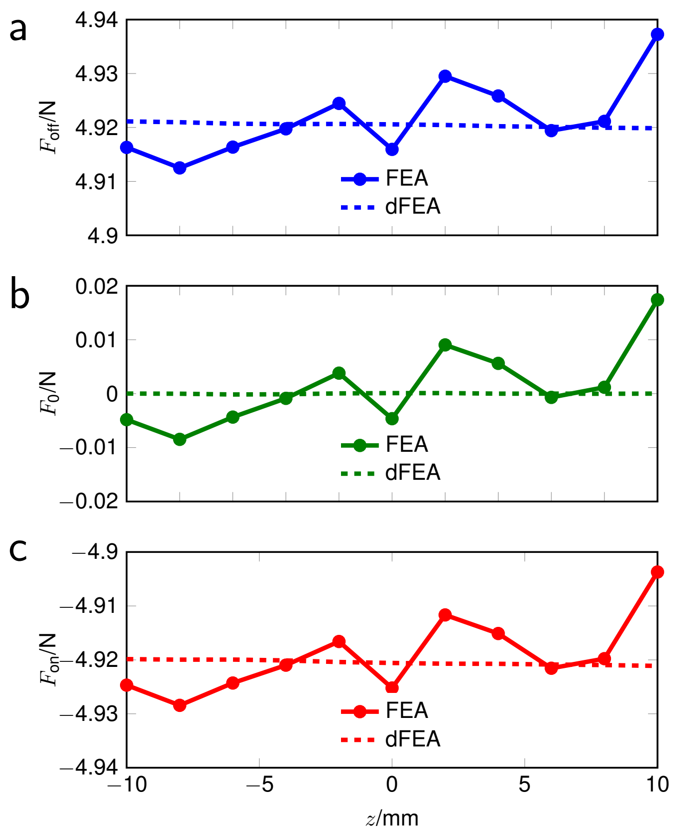

FIG. 2. Simulation results of the magnetic force of a coil in a magnetic field with different currents: (a) $I = I_{\mathrm{off}}$, (b) $I = 0$ and (c) $I = I_{\mathrm{on}}$. Note that this example is the case with $\chi = -0.01$. The solid lines are obtained from FEA calculations, while in (a) and (c) the dashed lines are differential signals, $F_{\mathrm{off}} - F_0$ and $F_{\mathrm{on}} - F_0$. The dashed line in (b) is half the difference of the two forces, $(F_{\mathrm{off}} - F_{\mathrm{on}})/2$. In order to plot it in the same scale the mean of 4.92 N has been subtracted.

the largest one about 1000 times the size of the nominal susceptibility.

---

[1] S Li, F Bielsa, M Stock, A Kiss, and H Fang. Coil-current effect in kibble balances: analysis, measurement, and optimization. *Metrologia*, 55(1):75–83, 2017.

[2] Hao Fang, Franck Bielsa, Shisong Li, Adrien Kiss, and Michael Stock. The BIPM Kibble balance for realizing the kilogram definition. *Metrologia*, 57:045009, 2020.

[3] H Ahmedov, N Babayiğit Aşkın, B Korutlu, and R Orhan. Preliminary Planck constant measurements via UME oscillating magnet kibble balance. *Metrologia*, 55(3):326–333, 2018.

[4] Ian A Robinson, James Berry, Christopher Bull, Stuart Davidson, Charles Jarvis, Peter Lovelock, Christopher Lucas, Jeannie Urquhart, Emily Webster, and Perdi Williams. Developing the next generation of NPL Kibble balances. In *2018 Conference on Precision Electromagnetic Measurements (CPEM 2018)*, pages 1–2. IEEE, 2018.

[5] Shisong Li, Franck Bielsa, Michael Stock, Adrien Kiss, and Hao Fang. A permanent magnet system for Kibble balances. *Metrologia*, 54(5):775–783, 2017.
